# Consumption of Red Meat, but Not Cooking Oils High in Polyunsaturated Fat, Is Associated with Higher Arachidonic Acid Status in Singapore Chinese Adults

**DOI:** 10.3390/nu9020101

**Published:** 2017-01-31

**Authors:** Jowy Yi Hoong Seah, Gibson Ming Wei Gay, Jin Su, E-Shyong Tai, Jian-Min Yuan, Woon-Puay Koh, Choon Nam Ong, Rob M. van Dam

**Affiliations:** 1 Saw Swee Hock School of Public Health, Singapore 117549, Singapore; E0011458@u.nus.edu (J.Y.H.S.); gibson4690@gmail.com (G.M.W.G.); jin_su@nuhs.edu.sg (J.S.); e_shyong_tai@nuhs.edu.sg (E-S.T.); woonpuay.koh@duke-nus.edu.sg (W.-P.K.); 2 Department of Medicine, Yong Loo Lin School of Medicine, National University of Singapore (NUS) and National University Health System, Singapore 119228, Singapore; 3 NUS Graduate School for Integrative Sciences and Engineering, Singapore 117456, Singapore; 4 NUS Environmental Research Institute, National University of Singapore, Singapore 117411, Singapore; 5 Duke-NUS Medical School, Singapore 169857, Singapore; 6 Division of Cancer Control and Population Sciences, University of Pittsburgh Cancer Institute, Pittsburgh, PA 15232, USA; yuanj@upmc.edu; 7 Department of Epidemiology, Graduate School of Public Health, University of Pittsburgh, Pittsburgh, PA 15261, USA; 8 Department of Nutrition, Harvard School of Public Health, Boston, MA 02115, USA

**Keywords:** diet, polyunsaturated fatty acids, cooking oil, inflammation, cardiovascular disease, plasma fatty acid, omega-6 fatty acid, omega-3 fatty acid, biomarkers

## Abstract

High arachidonic acid (AA; 20:4*n*-6) status may have adverse effects on inflammation and risk of cardiovascular diseases. Concerns about high intake of *n*-6 polyunsaturated fatty acids (PUFAs) are based on the premise that endogenous conversion from linoleic acid (LA; 18:2*n*-6) is an important source of AA, but few population-based studies have investigated dietary determinants of AA status. In this study, we examined habitual food consumption in relation to plasma concentrations of AA and other PUFAs in population-based studies. We used cross-sectional data from 269 healthy, ethnic Chinese participants (25–80 years old) with contrasting intakes of fish and red meat from the Singapore Prospective Study Program and 769 healthy participants (44–74 years old) from the Singapore Chinese Health Study as a validation set. Multivariable linear regression was used to examine PUFA intake (% energy) and food sources of PUFA (fish, red meat, poultry, soy and cooking oils) in relation to plasma PUFAs (AA, LA, dihomo-gamma-linolenic acid (DGLA; 20:3*n*-6), alpha-linolenic acid (ALA; 18:3*n*-3), eicosapentaenoic acid (EPA; 20:5*n*-3), and docosahexaenoic acid (DHA; 22:6*n*-3)) concentrations. Higher intake of red meat was associated with higher plasma AA concentrations. High intake of PUFA or PUFA-rich oils was associated with higher plasma ALA but not with plasma AA. Higher intakes of soy were associated with higher ALA and fish with higher DHA and EPA concentrations. These associations were statistically significant (*p* < 0.05) in both studies. Red meat consumption, but not PUFA or PUFA-rich cooking oil, was associated with circulating AA suggesting that intake of pre-formed AA rather than LA is an important determinant of AA status. A diet high in fish, soy products and polyunsaturated cooking oil, and low in red meat may be associated with an optimal plasma profile of PUFA in this Chinese population.

## 1. Introduction

The role of *n*-6 polyunsaturated fatty acids in cardiovascular health and inflammation is controversial. A high polyunsaturated fat (PUFA) intake, of which the greatest proportion is the *n*-6 PUFA linoleic acid (LA; 18:2*n*-6), may lower cardiovascular heart disease (CVD) risk by lowering low-density lipoprotein concentrations [1,2,3] and blood pressure [4]. In prospective observational studies, dietary LA intake was inversely associated with risk of coronary artery disease [5]. However, potential detrimental effects of LA have also been suggested [6]. In the body, LA can be converted into arachidonic acid (AA; 20:4*n*-6) through desaturation and elongation reactions [7]. Arachidonate-derived prostaglandins (PGE_2_) and leukotrienes have been implicated in CVD [8,9]. Increased AA content in adipose tissue has also been associated with a higher risk of coronary artery disease [10]. However, AA is also the precursor to lipoxins that are involved in reducing inflammation [11]. Dihomo-gamma-linolenic acid (DGLA; 20:3*n*-6) is an elongation product of gamma-linolenic acid and direct precursor of AA that may also have an independent role as an inflammation mediator [12]. 

Higher intakes of the marine-originated long-chain *n*-3 eicosapentaenoic acid (EPA; 20:5*n*-3) and docosahexaenoic acid (DHA; 22:6*n*-3) were associated with less inflammation [13] and a lower risk of fatal and nonfatal coronary artery disease in prospective cohort studies [14,15]. Results for the effects of EPA and DHA on CVD risk in clinical trials have been inconsistent [16,17]. Less is known about the short-chain plant-derived alpha-linolenic acid (ALA; 18:3*n*-3); while some epidemiological studies point to its cardio-protective effects, especially from sudden death [18,19], others did not suggest beneficial effects [20,21]. ALA can be converted to EPA and DHA through desaturation and elongation, although conversion appears to be tightly regulated [22]. ALA may also affect CVD risk independent from EPA and DHA [23].

It has been postulated that a high ratio of *n*-6 to *n*-3 PUFA in the diet may increase risk of inflammatory diseases and CVD [24] as well as other health conditions such as obesity and non-alcoholic liver disease [25]. These are based on the premise that endogenous conversion from LA is an important source of AA. Since pre-formed AA is naturally found in meat [26], AA-rich foods may also increase circulating AA concentrations [27]. Most of the available evidence on dietary determinants of AA is from small-scale dietary interventions conducted in the Western population [28,29,30,31]. We therefore examined PUFA intake and food sources of PUFAs in relation to the six PUFAs (LA, ALA, DGLA, AA, EPA, DHA) that may be involved in inflammation or CVD in two population-based studies in ethnic Chinese residing in Singapore.

## 2. Subjects and Methods

### 2.1. Singapore Prospective Study Program (SP2)

#### 2.1.1. Study Population and Design

We selected 269 participants from the Singapore Prospective Study Program (SP2), a population-based study conducted between 2004 and 2007 in Singapore. The methods of this study have previously been described in detail [32]. In brief, all participants in the study took part in four previous representative population-based studies with over-sampling of ethnic minority groups conducted in Singapore. The participants of the earlier surveys were contacted for an interview using standardized questionnaires on lifestyle factors and medical history at their homes. Following the interviews, all participants were invited to attend a health examination for additional tests and collection of blood samples. For the purpose of our current study, we focused on the 3425 participants of Chinese ethnicity. We excluded participants who were more than 80 years old (*n* = 13), who were smokers (*n* = 375), who had missing age (*n* = 3) or BMI (*n* = 5) or who had reported extreme total energy intakes (<1000 kcal or >3000 kcal for women and <1500 kcal or >3500 kcal for men per day; *n* = 575) resulting in 2458 remaining participants. The participants might be excluded for one or more reasons. The large cohort allowed us to use stringent selection criteria to minimize confounding and errors in reporting. We selected three groups based on pre-defined dietary characteristics: (1) A *High Fish* intake group (fish ≥ 150 g/2000 kcal and red meat < 100 g/2000 kcal; *n* = 112 females and 62 males); (2) A *High Red Meat* intake group (red meat ≥ 100 g/2000 kcal and fish < 150 g/2000 kcal; *n* = 118 females and 66 males); and (3) A *Low Fish and Red Meat* intake group (fish < 40 g/2000 kcal and red meat < 25 g/2000 kcal; *n* = 97 females and 51 males). The cutoffs of 150 g/2000 kcal for fish and 100 g/2000 kcal for red meat were chosen to reflect daily consumption of at least one standard portion and to achieve maximum contrast in intakes between the three dietary groups. As our SP2 study population had a higher intake of fish than red meat, we could select a higher cutoff for fish intake than for red meat intake. To ensure an equal sex distribution in each dietary group, we randomly sampled 50 females and 50 males from each dietary group, amounting to 300 participants. However, 31 participants had insufficient blood samples remaining for the laboratory measurements resulting in a final number of 269 participants for the current analysis.

Ethics approval was obtained from the institutional review boards of the National University of Singapore and Singapore General Hospital. The project identification for the NUS IRB was NUS 1650 for this sub-study. Informed consent was obtained from all participants before the study was conducted.

#### 2.1.2. Assessment of Diet and Covariates

A trained interviewer ascertained dietary information over the past month using a 159-item semi-quantitative food frequency questionnaire (FFQ). For each of the food items, the respondent was asked to report intake as frequency per day, per week, per month, or rarely/never. The nutrient and energy intakes for the participants were subsequently calculated by the Singapore Health Promotion Board using an in-house database with information on energy and nutrient values of local foods. The amount of energy and each of the nutrients contributed by each food item was computed based on its frequency of consumption, weight of the food item consumed and its nutrient composition. The FFQ was validated against three 24-hour recalls and correlation coefficients for energy or nutrient intakes assessed with the FFQ and 24-hour recalls ranged from 0.46 to 0.58 [33]. We also calculated the consumption of key food sources of omega-3 and omega-6 PUFA (fish, red meat, poultry and soy) taking into account both intakes as a main ingredient and as part of mixed dishes based on standard recipes. The type of cooking oil (blended vegetable oil, polyunsaturated oil, monounsaturated oil, saturated oil) used was also considered.

Physical activity in the leisure time, occupational, household, and transport domain were assessed using a validated questionnaire [34] and metabolic equivalent task (MET)-hours per week were calculated [35]. The consumption of alcoholic drinks was assessed in the FFQ and converted to grams of alcohol per day.

#### 2.1.3. Physical Examination and Blood Collection

Height was measured without shoes by using a wall-mounted stadiometer. Participants were instructed to wear light clothing and to remove any heavy objects such as keys before their weight was taken using a digital scale [32]. BMI was calculated by taking the weight (kg) of a participant divided by the square of his height (m^2^).

Participants were examined in the morning after a 10-hour overnight fast. Venous blood was drawn and collected in plain and fluoride oxalate tubes and was stored at 4 °C before processing. The maximum time of storage prior to processing was 4 h. Thereafter, the samples were stored at −80 °C [32].

### 2.2. Singapore Chinese Health Study (SCHS)

#### 2.2.1. Study Population and Design

The Singapore Chinese Health Study (SCHS) is a population-based, prospective cohort of lifestyle and chronic disease risk. It enrolled 63,257 ethnic Chinese men and women aged 45–74 years between 1993 and 1998. All participants were residents of government-built housing estates that represented 86% of where the Singapore population resided during the enrollment period. A trained interviewer collected information on baseline diet, lifestyle and medical history from each consenting participant with the use of a structured questionnaire. For the current analysis, we used data from the controls (*n* = 769) of a case-control study of acute myocardial infarction nested in SCHS. The design of the study has been described in detail previously [36]. All participants gave informed consent and the institutional review board at the National University of Singapore approved the study. The project identification for the NUS IRB was NUS 1058 for this sub-study.

#### 2.2.2. Assessment of Diet and Covariates

Diet was assessed using an interviewer-administered validated 165-item semi-quantitative FFQ. Respondents were asked to report intakes from eight different frequencies (ranging from never or hardly ever to ≥2 times per day). Type of cooking oil (lard, palm/blended oil, corn oil, peanut oil, soybean oil or sesame oil) was also assessed. The Singapore Food Composition Table was developed in conjunction with this cohort. The intake of energy, nutrients and food groups was calculated based on the FFQ. The FFQ was subsequently validated against a series of 24-hour dietary recalls among a subcohort of SCHS participants, and the correlation coefficients of energy or nutrients from this validation study ranged between 0.24 and 0.79 [37]. 

Participants were asked to self-report their height and weight in the questionnaire. BMI was calculated by taking the weight (kg) of a participant divided by the square of his/her height (m^2^) [38]. The respondents were also asked to report the frequency of moderate, vigorous and strenuous physical activity. These were converted to approximate MET-hours per week according to the five-level classification of physical activity based on exercise intensity [39].

#### 2.2.3. Blood Collection

During April 1994 to December 1999, a random 3% of the study participants donated blood for research. Between January 2000 and April 2005, we extended the biospecimen collection to 32,543 participants, which represented a consent rate of about 60% of surviving cohort participants at that time. The tubes containing the blood specimens were kept on ice during transport until they reached the laboratory, in which they were then separated into their various components (plasma, serum, red blood cells and buffy coat). The biospecimens were stored in −80 °C freezers.

### 2.3. Measurement of Plasma Fatty Acids

Plasma fatty acids were measured in the same laboratory using the same protocol for the SP2 and SCHS study. Gas chromatography-tandem mass spectrometry conducted on an Agilent 7890GC system equipped with a 7001B QQQ triple quadruple mass detector (Agilent, Santa Clara, CA, USA) and an autosample injector was used to measure the plasma fatty acids of the participants. Total fatty acids including both free and esterified (TGs, phospholipids, cholesterol esters) fractions were measured. There were 19 plasma fatty acids measured including the six PUFAs that are the focus of our study: (LA, ALA, DGLA, AA, EPA, DHA). The within-batch coefficients of variation (CVs) ranged from 3.7% to 8.1%, whereas the between-batch CVs ranged from 7.8% to 16.8%.

### 2.4. Statistical Analysis

The six plasma fatty acids studied were expressed as a percentage of plasma total fatty acids. PUFA intake, as well as *n*-6 and *n*-3 intake (both available only for SCHS), were expressed as a percentage of total energy. The *n*-6/*n*-3 intake was obtained by dividing *n*-6 by *n*-3 intake. These six plasma fatty acids and nutrient intake variables were then log-transformed. Any values not within mean ± 4 standard deviation of the log-transformed variables were identified as outliers and truncated to improve normality. Dietary variables (red meat, fish, soy, poultry) were treated as continuous variables, whereas cooking oil use was modeled categorically with blended vegetable oil (SP2) or palm/blended oil (SCHS) as the reference group. For the cooking oil analysis in SCHS, we restricted participants to those using cooking oil at least once per day, resulting in 592 participants.

For the SP2 study, the Pearson’s chi-squared test (for categorical variables) and ANOVA (for continuous variables) were used to compare population characteristics between the three groups with distinct diets. ANOVA was also used to compare plasma fatty acids amongst the three dietary groups. The first model presents the unadjusted means and the second model presents means (least squared means) adjusted for sex (male/female), age (years), BMI (kg/m^2^), energy intake (kcal), waist circumference (cm), physical activity (MET-hours/week) and alcohol (g/day).

Multivariable linear regression analysis was used to model associations between selected dietary variables (fish, red meat, poultry, soy expressed per 50 g/day increment and PUFA and *n*-6 PUFA intake expressed as energy %) and plasma fatty acids. We also conducted the analysis for type of cooking oil used with “blended vegetable oil” (SP2) and “palm/blended oil” (SCHS) as the reference. Two models are presented: the first model adjusted for sex (male/female) and age (years) and the second model additionally adjusted for BMI (kg/m^2^), energy intake (kcal), waist circumference (cm, only available for SP2), physical activity (MET-hours/week), alcohol (g/day) and consumption of fish, red meat, and poultry (if these were not the exposure of interest). For the SCHS study, we additionally tested for interactions by sex, age (median age: ≥60 and <60 years) and overweight status (using the Asian criteria of ≥23 kg/m^2^) by including a multiplicative term with sex, age or overweight status as a binary variable and dietary variables (fish, red meat, poultry, soy, PUFA, *n*-6 PUFA) as continuous variables in fully adjusted models. The modest sample size of the SP2 study (*n* = 269) and the modest numbers of users of different cooking oils, did not allow for such a stratified analysis to be meaningful as the statistical power would be too limited. STATA Software version 14 was used for all statistical analyses and *p*-values < 0.05 were considered statistically significant.

## 3. Results

### 3.1. Characteristics of the Study Populations

Characteristics of the 269 participants from SP2 were examined according to three dietary groups: “High Fish,” “High Red Meat,” and “Low Fish, Low Red Meat” (Table 1). As a result of the selection criteria, there was an expected large difference in fish and red meat intake between these groups. There were also significant differences in poultry intake (higher in the High Red Meat group) and soy intake (higher in the Low Fish, Low Red Meat group). No differences were observed with regard to non-dietary characteristics and cooking oil use, with the exception of a lower waist circumference among participants of the Low Fish, Low Red Meat group compared with groups with higher fish or red meat consumption. The baseline characteristics of the SCHS participants are shown in Supplementary Table S1. 

### 3.2. Plasma Polyunsaturated Fat (PUFA) Concentrations by Fish and Red Meat Consumption

Plasma PUFA concentrations differed significantly between the three dietary groups (Table 2). The High Fish group had the highest plasma concentration of EPA and DHA; the High Red Meat group had the highest AA concentrations; and the Low Fish, Low Red Meat group had the lowest concentration of EPA, DHA, and AA. In contrast, the Low Fish, Low Red Meat group had the highest plasma concentrations of LA and DGLA and High Red Meat group the lowest concentration of these fatty acids (Table 2). These associations remained significant after adjustments for lifestyle factors and other dietary variables. ALA concentrations did not differ substantially between the three dietary groups.

In the independent SCHS population, we examined consumption of red meat, fish, poultry and soy as continuous variables in relation to plasma PUFA concentrations (Table 3).

Higher intake of red meat was significantly associated with higher plasma AA and lower plasma ALA concentrations. Higher fish intake was associated with higher concentrations of long-chain *n*-3 EPA and DHA and lower concentrations of DGLA. Higher soy intake was associated with higher plasma ALA. While higher poultry consumption was associated with higher EPA and DHA concentrations, the association with EPA was attenuated after further adjustments for lifestyle factors.

### 3.3. PUFA Intake and Cooking Oil Use in Relation to Plasma PUFA

We also examined PUFA intake and cooking oil use in relation to PUFA concentrations (Table 4). Higher PUFA intake (energy %) was associated with higher plasma LA and ALA concentrations in both studies. In contrast, no such association was observed between PUFA intake and plasma AA concentrations. The associations observed for *n*-6 PUFA intake (only available for SCHS) were similar to those for total PUFA intake, as intakes were highly correlated (*r* = 0.997). The *n*-6/*n*-3 ratio (available only for SCHS) was not significantly associated with plasma AA, DGLA, EPA or DHA (results not shown).

In the SP2 study, the use of cooking oils high in polyunsaturated or monounsaturated fatty acids was associated with higher concentrations of plasma ALA compared with the use of blended oils that are typically high in palm oil. In the SCHS study, where the dietary questionnaire included more detailed categories for cooking oil, soybean oil use was significantly associated with higher plasma ALA. The type of cooking oil used was not associated with DGLA with the exception of peanut oil in the SCHS. The choice of cooking oil was not associated with higher AA concentrations in either study.

### 3.4. Effect Modification by Sex, Age and Overweight Status

We also examined possible effect modification of the association between dietary intakes and plasma fatty acids by age, sex, and overweight status in the SCHS study (Supplementary Tables S2–S4). Results for red meat, fish and PUFA intakes in relation to plasma fatty acids were consistent across age, sex, and overweight status. We did observe associations between soy and poultry intakes and plasma fatty acids that were restricted to population subgroups. In participants younger than 60 years, soy intake was significantly associated with lower AA levels while this was not observed in older participants (P interaction < 0.05). Furthermore, poultry intake was significantly associated with higher circulating DHA levels in women, but not in men (P interaction < 0.01). Finally, poultry and soy intakes were significantly associated with higher circulating EPA levels in overweight participants, but not in leaner participants (all P interaction < 0.05).

## 4. Discussion and Conclusions

In two population-based studies of ethnic Chinese adult men and women, we observed a consistent association between higher consumption of red meat and higher arachidonic acid (AA) plasma concentrations. High intake of polyunsaturated fat (PUFA) was associated with higher plasma alpha-linolenic acid (ALA) and linoleic acid (LA) concentrations, but not with plasma AA. Similarly, use of cooking oils high in PUFA was associated with higher ALA, but not with higher AA concentrations. Higher intakes of soy were associated with higher ALA and fish with higher docosahexaenoic acid (DHA) and eicosapentaenoic acid (EPA) concentrations. 

We observed an association between high consumption of red meat and higher AA concentrations in both studies, and this is consistent with short-term intervention studies that observed an increase in AA concentrations after participants consumed foods containing pre-formed AA [28,29,30,31]. The AA content of red meat ranges from 21 mg to 180 mg for 100 g of meat, and varies with type, source, method of cooking and portions of the meat [40,41]. Additionally, we found no association between use of oils high in PUFA, total PUFA intake, or *n*-6 PUFA intake and AA concentrations. Our findings are in line with a meta-analysis of feeding trials where varying the LA intake, the major *n*-6 PUFA, of participants from −80% to +600% from baseline was not associated with changes in circulating AA concentrations [27]. Although fish is also a source of AA, we did not observe an association between fish intake and elevated AA concentrations in the Singapore Chinese Health Study (SCHS). The lack of association is likely due to the large differences in AA content among different types of fish, and our food frequency questionnaire (FFQ) for both studies did not capture the specific type of fish consumed [42]. It is possible that participants in the two studies exhibited different preferences for types of fish.

Our results further suggest that a diet with higher amounts of fish, soy products and polyunsaturated oil, especially soybean oil, is associated with higher plasma concentrations of *n*-3 PUFAs. Consistent with our findings, the association between fish consumption and higher EPA and DHA concentrations is well-documented [43]. The short-chain *n*-3 PUFA ALA is a minor component in soy and soybean oil and our findings suggest that these are indeed important determinants of ALA concentrations in ethnic Chinese populations.

A high intake of *n*-6 PUFA or *n*-6/*n*-3 ratio has been postulated to increase inflammation and cardiovascular disease (CVD) risk, with the current Western diet having a high ratio of 15:1 to 20:1 [24]. These arguments are rooted in the premise that endogenous conversion of LA contributes substantially to circulating AA concentrations. The endogenous conversion of ALA to EPA and subsequently DHA involves the same elongase and desaturase enzymes, resulting in substrate competition between the *n*-3 and *n*-6 pathways [22,44]. As both LA and ALA compete for delta-6 desaturase, the enzyme involved in the rate-limiting step, a high *n*-6/*n*-3 ratio intake may theoretically inhibit the conversion of ALA into EPA and DHA while favouring the increase in AA concentrations [22,24]. AA is a substrate for various eicosanoids, many of which may have detrimental effects on inflammation and CVD [8,9] whereas resolvins and protectins derived from long-chain *n*-3 EPA and DHA have been suggested to be anti-inflammatory and cardioprotective [45,46,47,48]. However, the lack of association between *n*-6 PUFA intake or its major food sources and plasma AA concentrations in our results do not support the hypothesis that endogenous conversion contributes significantly to AA concentrations. Similarly, there was no association between foods rich in ALA such as soy products and plasma EPA or DHA, suggesting that endogenous conversion of ALA is not an important determinant of long-chain *n*-3 PUFA status in this population. The low efficiency of conversion of both short-chain *n*-3 and *n*-6 fatty acids to its long-chain fatty acid counterparts is consistent with results from previous studies [22,26]. Our results suggest that dietary intake of pre-formed long-chain fatty acids from fish (for EPA and DHA) and red meat (for AA) are key determinants of plasma concentrations of these fatty acids. Furthermore, we did not observe effect modification by sex, age or overweight status in the association between fish consumption and EPA and DHA concentrations, or that of red meat intake and AA concentrations, with the different strata showing similar effect estimates. The consistent lack of association between PUFA or *n*-6 PUFA intake and circulating AA concentrations across different strata also adds strength to our conclusions.

While we observed some differences in the association between soy and poultry intake and plasma AA, EPA and DHA levels by sex, age and overweight status, these differences were inconsistent and may reflect behavioural dietary patterns or chance findings. For example, poultry consumption may have been associated with a preference for fatty rather than leaner types of fish*.* This possible confounding variable could explain why poultry intake was associated with higher DHA levels but not EPA levels*.* Additional studies are required to confirm the observed interactions.

A strength of our SP2 study was the stringent cut-offs we used for the selection of the 269 participants, resulting in large differences in fish and red meat intake. In addition, both Singapore Prospective Study Program (SP2) and SCHS used detailed validated interviewer-administered FFQs and collected information on a wide range of potential confounders. We selected key lifestyle factors that may independently influence plasma fatty acid composition as potential confounders based on evidence from the scientific literature *a priori*, and included these in the fully adjusted models [49]. Our study also included persons from the general population rather than specific clinical population samples and this would limit variation in metabolic factors that might influence plasma fatty acid composition. Furthermore, confirmation of our results in an independent study population reduces the probability of statistically significant associations due to chance. Our study also had several potential limitations that should be considered. First, blood fatty acid concentrations can be affected by changes in the recent diet [50] and this may have weakened the observed associations between habitual dietary intakes and plasma fatty acids in our study. However, concentrations of fatty acid biomarkers tend to have reasonably good long-term reproducibility in population-based studies including those in other Asian populations [51]. Second, a weakness common to all observational studies is the possibility of residual confounding in diet and lifestyle that we cannot fully exclude. Third, measurement error is inevitable as our assessment of diet was based on self-reports, although this would have more likely weakened than strengthened the observed associations. Fourth, it is difficult to infer causality with cross-sectional data. However, it is unlikely that the participants were aware of their plasma fatty acid profile and that this would have affected their dietary choices. Finally, we also urge caution in generalizing our findings to populations of a different ethnicity. 

Our results do not support concerns regarding a high PUFA intake that are based on the premise of endogenous conversion of LA to AA. Consumption of red meat, a source of pre-formed AA, instead of dietary PUFA or cooking oils high in PUFA was associated with higher circulating AA concentrations. In sum, our findings suggest that a diet with higher amounts of fish, soy products and polyunsaturated oil, along with lower amounts of red meat might be optimal to achieve a plasma polyunsaturated fatty acids profile that has been associated with lower inflammation and risk of CVD.

## Figures and Tables

**Table 1:** Characteristics of 269 Singapore Prospective Study participants with high red meat, high fish, or low red meat and fish consumption.

Variable	All		Low Fish, Low Red Meat		High Fish		High Red Meat		*p*-Value
	*N* = 269		*N* = 86		*N* = 89		*N* = 94		
	Mean	SD	Mean	SD	Mean	SD	Mean	SD	
Age (years)	51.4	12.2	50	11.2	52.1	11.7	52.1	13.3	0.401
BMI (kg·m^−2^)	23.2	3.9	22.5	3.8	23.7	3.9	23.2	3.9	0.112
Waist circumference (cm)	82.3	11.3	79.8	11.7	83.8	11.3	83.1	10.7	0.047
Energy intake (kcal)	2008.9	550.9	1965.5	534.9	1946.7	554.9	2107.3	553.5	0.097
Total physical activity (MET-hours/week) ^1^	114.4	103.8	108.2	76	107.2	77.5	127	140.9	0.347
Alcohol (g/day)	14.4	69.8	8.3	32.1	11.3	38.2	22.8	107.8	0.336
Red meat (g/2000 kcal)	68.6	62.4	14.1	8.4	44.7	24.4	141	44.9	<0.001
Fish (g/2000 kcal)	100	77.9	24.6	14.4	193.2	47.6	80.8	35.2	<0.001
Soy (g/2000 kcal)	16.5	17.2	21.4	23.5	15.0	14.1	13.4	11.1	0.005
Poultry (g/2000 kcal)	42.8	32.9	26.7	22.9	42.5	28.2	57.9	37.6	<0.001
	*N*	%	*N*	%	*N*	%	*N*	%	
Sex	-		-	-	-	-	-	-	0.868
Male	134	49.8%	41	30.6%	46	34.3%	47	35.1%	
Female	135	50.2%	45	33.3%	43	31.9%	47	34.8%	
Type of cooking oil used ^2^									0.861
Blended vegetable oil	99	36.8%	31	31.3%	30	30.3%	38	38.4%	
Polyunsaturated oil	78	29.0%	26	33.3%	24	30.8%	28	35.9%	
Monounsaturated oil	84	31.2%	27	32.1%	31	36.9%	26	31.0%	

^1^ Refers to Metabolic Equivalent of Task: hours per week. ^2^ Blended oil is palm oil mixed with vegetable oils high in mono- or polyunsaturated fat. No participants chose cooking oil high in saturated fat (lard, ghee, tallow, butter, margarine, coconut oil, palm oil); eight participants (3.0%) answered ‘Not Applicable’ to the question of what cooking oil/fat was used for meals prepared at home.

**Table 2:** Plasma fatty acid composition (weight %) of 269 Singapore Prospective Study Program participants with high red meat, high fish, or low red meat and fish consumption.

Fatty Acid	All		Low Fish, Low Red Meat		High Fish		High Red Meat		*p*-Value ^1^
	*N* = 269		*N* = 86		*N* = 89		*N* = 94		
	Mean	SD	Mean	SD	Mean	SD	Mean	SD	
18:2*n*-6 (LA) ^2^	50.05	5.36	51.54 ^a^	5.52	49.72 ^b^	4.26	48.99 ^b^	5.87	0.004
Adjusted means ^3^	-	-	51.05 ^a^	-	50.00 ^b^	-	49.17 ^b^	-	<0.001
18:3*n*-3 (ALA)	0.22	0.09	0.22	0.1	0.24	0.09	0.21	0.09	0.201
Adjusted means	-	-	0.22	-	0.24	-	0.22	-	0.531
20:3*n*-6 (DGLA)	0.74	0.32	0.80 ^a^	0.35	0.67 ^b^	0.27	0.76 ^a,b^	0.33	0.022
Adjusted means	-	-	0.83 ^a^	-	0.66 ^b^	-	0.74 ^a,b^	-	0.026
20:4*n*-6 (AA)	6.39	2.13	5.56 ^a^	1.9	6.65 ^b,c^	1.97	6.92 ^b,c^	2.26	<0.001
Adjusted means	-	-	5.60 ^a^	-	6.70 ^b,c^		6.84 ^b,c^	-	<0.001
20:5*n*-3 (EPA)	0.3	0.19	0.23 ^a^	0.13	0.38 ^b^	0.24	0.29 ^c^	0.16	<0.001
Adjusted means	-	-	0.23 ^a^	-	0.38 ^b^	-	0.29 ^c^	-	<0.001
22:6*n*-3 (DHA)	1.02	0.46	0.73 ^a^	0.33	1.27 ^b^	0.47	1.07 ^c^	0.39	<0.001
Adjusted means	-	-	0.73 ^a^	-	1.26 ^b^	-	1.07 ^c^	-	<0.001

^1^ The *p*-values are based on ANOVA. Values with different assigned superscript-letters are significantly different from each other. ^2^ Fatty acid abbreviations: LA (linoleic acid; 18:2*n*-6), ALA (alpha-linolenic acid; 18:3*n*-3), DGLA (dihomo-gamma-linolenic acid; 20:3*n*-6), AA (arachidonic acid; 20:4*n*-6), EPA (eicosapentaenoic acid; 20:5*n*-3), DHA (docosahexaenoic acid; 22:6*n*-3). ^3^ Adjusted for sex, age, body mass index, waist circumference, physical activity, total energy intake and alcohol consumption.

**Table 3:** Association between consumption of meat and meat alternatives and plasma fatty acid composition (weight %) of the Singapore Chinese Health Study participants (*n* = 769) ^1^.

Dietary Variables		Fatty Acid (%)					
		LA ^2^	ALA	DGLA	AA	EPA	DHA
Fish (per 50 g)	β_1_ (SE)	0.318 (0.251)	0.009 (0.012)	−0.038 (0.017) *	−0.025 (0.095)	0.085 (0.021) **	0.468 (0.077) **
	β_2_ (SE)	0.338 (0.254)	0.012 (0.012)	−0.043 (0.018) *	−0.063 (0.096)	0.089 (0.022) **	0.479 (0.079) **
Red meat (per 50 g)	β_1_ (SE)	0.071 (0.395)	−0.057 (0.018) **	0.012 (0.027)	0.428 (0.018) **	−0.003 (0.034)	−0.052 (0.124)
	β_2_ (SE)	−0.083 (0.411)	−0.068 (0.019) **	0.020 (0.028)	0.500 (0.155) **	−0.047 (0.035)	−0.260 (0.127)
Poultry (per 50 g)	β_1_ (SE)	0.150 (0.442)	0.014 (0.020)	−0.007 (0.030)	0.060 (0.166)	0.075 (0.038) *	0.425 (0.138) **
	β_2_ (SE)	−0.104 (0.458)	0.029 (0.021)	−0.003 (0.032)	−0.010 (0.173)	0.068 (0.039)	0.454 (0.141) **
Soy (per 50 g)	β_1_ (SE)	0.096 (0.091)	0.012 (0.004) **	0.001 (0.006)	−0.069 (0.034) *	0.008 (0.008)	0.003 (0.029)
	β_2_ (SE)	0.031 (0.091)	0.011 (0.004) *	0.001 (0.006)	−0.041 (0.034)	0.005 (0.008)	−0.002 (0.028)

^1^ β (SE) are regression coefficients (standard errors) from linear regression analysis. Values for the β coefficients represent the percentage change in plasma fatty acid composition for every 50 g per day increment in fish, red meat, poultry and soy consumption. β_1_: model adjusted for sex and age; β_2_: model additionally adjusted for body mass index, physical activity, total energy intake, alcohol consumption, and other meats or meat alternatives (fish, red meat, poultry, and soy). * *p*-value < 0.05; ** *p*-value < 0.01. ^2^ Fatty acid abbreviations: LA (linoleic acid; 18:2*n*-6), ALA (alpha-linolenic acid; 18:3*n*-3), DGLA (dihomo-gamma-linolenic acid; 20:3*n*-6), AA (arachidonic acid; 20:4*n*-6), EPA (eicosapentaenoic acid; 20:5*n*-3), DHA (docosahexaenoic acid).

**Table 4:** Association between polyunsaturated fat (PUFA) and cooking oil intake and plasma fatty acid composition (weight %) of Singapore Prospective Study Program (SP2) and Singapore Chinese Health Study (SCHS) participants ^1^.

Dietary Variables		Fatty Acid (%)					
		LA ^2^	ALA	DGLA	AA	EPA	DHA
**SP2 (*N* = 269)**							
PUFA intake (en %)	β_1_ (SE)	0.284 (0.113) *	0.008 (0.002) **	−0.013 (0.07)	−0.032 (0.046)	0.000 (0.008)	0.009 (0.010)
	β_2_ (SE)	0.259 (0.106) *	0.008 (0.002) **	−0.013 (0.07)	−0.007 (0.044)	0.000 (0.004)	0.011 (0.010)
Polyunsaturated oil (reference: blended vegetable oil)	β_1_ (SE)	1.044 (0.786)	0.044 (0.014) **	−0.050 (0.048)	−0.116 (0.324)	0.004 (0.029)	−0.004 (0.068)
	β_2_ (SE)	0.982 (0.744)	0.045 (0.014) **	−0.059 (0.046)	−0.066 (0.297)	0.009 (0.027)	0.022 (0.057)
Monounsaturated oil (reference: blended vegetable oil)	β_1_ (SE)	0.630 (0.771)	0.042 (0.013) **	−0.049 (0.047)	−0.269 (0.317)	0.008 (0.028)	0.022 (0.067)
	β_2_ (SE)	0.389 (0.740)	0.038 (0.014) **	−0.037 (0.046)	−0.137 (0.296)	−0.003 (0.027)	0.002 (0.057)
**SCHS (*N* = 769)**							
PUFA intake (en %)	β_1_ (SE)	0.278 (0.063) **	0.017 (0.003) **	−0.003 (0.005)	−0.046 (0.024)	0.009 (0.005) *	0.032 (0.019)
	β_2_ (SE)	0.250 (0.063) **	0.017 (0.003) **	−0.009 (0.005) *	−0.042 (0.024)	0.009 (0.005) *	0.036 (0.019)
*n*-6 PUFA intake (en %)	β_1_ (SE)	0.295 (0.067) **	0.019 (0.003) **	−0.003 (0.005)	−0.048 (0.025)	0.010 (0.005) *	0.032 (0.020)
	β_2_ (SE)	0.267 (0.067) **	0.018 (0.003) **	−0.009 (0.005)	−0.043 (0.025)	0.010 (0.005) *	0.036 (0.020)
Corn oil (reference: palm/blended oil)	β_1_ (SE)	0.743 (0.480)	0.048 (0.023) *	−0.006 (0.034)	0.061 (0.195)	0.057 (0.043)	0.125 (0.159)
	β_2_ (SE)	0.856 (0.479)	0.044 (0.024)	−0.018 (0.034)	0.117 (0.194)	0.061 (0.043)	0.128 (0.156)
Peanut oil (reference: palm/blended oil)	β_1_ (SE)	0.435 (0.471)	−0.010 (0.023)	−0.075 (0.034) *	−0.366 (0.191)	−0.076 (0.042)	0.004 (0.155)
	β_2_ (SE)	0.430 (0.466)	−0.012 (0.023)	−0.071 (0.033) *	−0.374 (0.189) *	−0.081 (0.042)	−0.053 (0.152)
Soybean oil (reference: palm/blended oil)	β_1_ (SE)	0.366 (0.661)	0.077 (0.032) *	−0.032 (0.047)	−0.118 (0.269)	−0.100 (0.059)	−0.268 (0.334)
	β_2_ (SE)	0.544 (0.655)	0.079 (0.032) *	−0.032 (0.047)	−0.173 (0.266)	−0.094 (0.059)	−0.273 (0.213)

^1^ β (SE) are regression coefficients (standard errors) from linear regression analysis. Values for the β coefficients represent the percentage change in plasma fatty acid composition for every 1% increment in total energy intake contributed by PUFA. Cooking oil use was modeled as a categorical variable with blended vegetable oil or palm oil as the control. β_1_: model adjusted for sex and age; β_2_: model adjusted for sex, age, BMI, waist circumference, total physical activity, total energy, alcohol and dietary variables (fish, red meat, poultry, soy) except in the case for PUFA intake. * *p*-value < 0.05; ** *p*-value < 0.01. ^2^ Fatty acid abbreviations: LA (linoleic acid; 18:2*n*-6), ALA (alpha-linolenic acid; 18:3*n*-3), DGLA (dihomo-gamma-linolenic acid; 20:3*n*-6), AA (arachidonic acid; 20:4*n*-6), EPA (eicosapentaenoic acid; 20:5*n*-3), DHA (docosahexaenoic acid).

## Supplementary Materials

The following are available online at www.mdpi.com/2072-6643/9/2/101/s1, Table S1: Characteristics and plasma fatty acid composition (weight %) of the Singapore Chinese Health Study participants (*n* = 769), Table S2: Association between consumption of meat and meat alternatives, polyunsaturated fat (PUFA) and plasma fatty acid composition (weight %) of the Singapore Chinese Health Study participants (*n* = 769) stratified by sex (*n* = 497 for men, *n* = 272 for women), Table S3: Association between consumption of meat and meat alternatives, polyunsaturated fat (PUFA) and plasma fatty acid composition (weight %) of the Singapore Chinese Health Study participants (*n* = 769) stratified by age (*n* = 367 for age < 60 years, *n* = 402 for age ≥ 60 years), Table S4: Association between consumption of meat and meat alternatives, polyunsaturated fat (PUFA) and plasma fatty acid composition (weight %) of the Singapore Chinese Health Study participants (*n* = 769) stratified by overweight status (*n* = 394 for BMI < 23 kg/m^2^, *n* = 375 for BMI ≥ 23 kg/m^2^).

## Abbreviations

AA	arachidonic acid
LA	linoleic acid
PUFA	polyunsaturated fatty acids / polyunsaturated fat
SP2	Singapore Prospective Study Program
SCHS	Singapore Chinese Health Study
DGLA	dihomo-gamma-linolenic acid
ALA	alpha-linolenic acid
EPA	eicosapentaenoic acid
DHA	docosahexaenoic acid
CVD	cardiovascular disease
FFQ	food frequency questionnaire
BMI	body mass index
CV	coefficient of variation
MET	metabolic task-equivalent
