# Supplementary material for: Consumption of Red Meat, but Not Cooking Oils High in Polyunsaturated Fat, Is Associated with Higher Arachidonic Acid Status in Singapore Chinese Adults
Source: Nutrients. 2017 Jan 31;9(2):101. doi: 10.3390/nu9020101 (PMC5331532; doi:10.3390/nu9020101)
Supplement: Supplementary file 1 [file nutrients-09-00101-s001.docx]

Supplementary Materials: Consumption of Red Meat, but Not Cooking Oils High in Polyunsaturated Fat, Is Asssociated with Higher Arachidonic Acid Status in Singapore Chinese Adults

Jowy Yi Hoong Seah, Gibson Ming Wei Gay, Jin Su, E-Shyong Tai, Jian-Min Yuan,
Woon-Puay Koh, Choon Nam Ong and Rob M. van Dam

**Table S1.** Characteristics and plasma fatty acid composition (weight %) of the Singapore Chinese Health Study participants (*n* = 769).

| **Variable** | ***N* = 769** | |
| --- | --- | --- |
|  | **Mean** | **SD** |
| Age (years) | 59.7 | 7.8 |
| BMI (kg m^−2^) | 22.8 | 2.9 |
| Energy intake (kcal) | 1584.2 | 521.6 |
| Total physical activity (MET-hours/week) ^1^ | 11.3 | 29.6 |
| Alcohol | 2.3 | 8 |
| Red meat (g/day) | 36.7 | 21.1 |
| Fish (g/day) | 64.9 | 33.1 |
| Soy (g/day) | 136.2 | 91.1 |
| Poultry (g/day) | 24.4 | 18.9 |
|  | ***N*** | **%** |
| Sex | 769 | 100.0% |
| Male | 497 | 64.6% |
| Female | 272 | 35.4% |
| Type of cooking oil used ^2^ | 592 | 100.0% |
| Palm / blended oil | 337 | 44.7% |
| Corn oil | 156 | 20.7% |
| Peanut oil | 163 | 21.6% |
| Soybean oil | 64 | 8.5% |
| Not applicable | 34 | 4.5% |
| **Fatty acid (%) ^3^** | **Mean** | **SD** |
| 18.2*n*6 (LA) | 36.47 | 4.59 |
| 18.3*n*3 (ALA) | 0.34 | 0.21 |
| 20.3*n*6 (DGLA) | 0.91 | 0.33 |
| 20.4*n*6 (AA) | 7.62 | 1.73 |
| 20.5*n*3 (EPA) | 0.52 | 0.39 |
| 22.6*n*3 (DHA) | 2.42 | 1.44 |

^1^ Refers to Metabolic Equivalent of Task: hours per week. These were approximated using the five-level classification of physical activity based on exercise intensity (37). ^2^ No participants chose 'lard, pork skin' to the question on what cooking oil/fat was used for meals prepared at home. ^3^ Fatty acid abbreviations: LA (linoleic acid; 18:2 *n −* 6), ALA (alpha-linolenic acid; 18:3 *n −* 3), DGLA (dihomo-gamma-linolenic acid; 20:3 *n −* 6), AA (arachidonic acid; 20:4 *n −* 6), EPA (eicosapentaenoic

acid; 20:5 *n −* 3), DHA (docosahexaenoic acid; 22:6 *n −* 3).

**Table S2.** Association between consumption of meat and meat alternatives, polyunsaturated fat (PUFA) and plasma fatty acid composition (weight %) of the Singapore Chinese Health Study participants (*n* = 769) stratified by sex (*n* = 497 for men, *n* = 272 for women) ^1^.

| **Dietary Variables** |  | **Fatty Acid (%)** | | | | | |
| --- | --- | --- | --- | --- | --- | --- | --- |
|  |  | **LA ^2^** | ***P*-Interaction** | **ALA** | ***P*-Interaction** | **DGLA** | ***P*-Interaction** |
| Fish (per 50 g) |  |  |  |  |  |  |  |
| Men | β_2_ (SE) | 0.450 (0.322) | 0.612 | 0.009 (0.016) | 0.833 | −0.040 (0.022) | 0.850 |
| Women | β_2_ (SE) | 0.094 (0.426) |  | 0.009 (0.017) |  | −0.043 (0.031) |  |
| Red meat (per 50 g) |  |  |  |  |  |  |  |
| Men | β_2_ (SE) | −0.153 (0.493) | 0.944 | −0.073 (0.024)** | 0.314 | 0.030 (0.033) | 0.939 |
| Women | β_2_ (SE) | 0.260 (0.763) |  | −0.042 (0.031) |  | 0.003 (0.055) |  |
| Poultry (per 50 g) |  |  |  |  |  |  |  |
| Men | β_2_ (SE) | 0.003 (0.578) | 0.571 | 0.019 (0.029) | 0.344 | −0.020 (0.039) | 0.409 |
| Women | β_2_ (SE) | −0.295 (0.760) |  | 0.043 (0.031) |  | 0.020 (0.055) |  |
| Soy (per 50 g) |  |  |  |  |  |  |  |
| Men | β_2_ (SE) | 0.069 (0.114) | 0.366 | 0.010 (0.006) | 0.909 | −0.004 (0.008) | 0.309 |
| Women | β_2_ (SE) | −0.047 (0.160) |  | 0.011 (0.006) |  | 0.007 (0.011) |  |
| PUFA intake (en %) |  |  |  |  |  |  |  |
| Men | β_2_ (SE) | 0.252 (0.109)* | 0.644 | 0.020 (0.005)** | 0.501 | −0.014 (0.007) | 0.128 |
| Women | β_2_ (SE) | 0.242 (0.158) |  | 0.015 (0.006)* |  | 0.001 (0.011) |  |
| *n −* 6 PUFA intake (en %) |  |  |  |  |  |  |  |
| Men | β_2_ (SE) | 0.272 (0.117)* | 0.601 | 0.022 (0.006)** | 0.353 | −0.015 (0.008) | 0.125 |
| Women | β_2_ (SE) | 0.250 (0.167) |  | 0.014 (0.007)* |  | 0.002 (0.012) |  |

**Table S2.** *Cont.*

| **Dietary Variables** |  | **Fatty Acid (%)** | | | | | |
| --- | --- | --- | --- | --- | --- | --- | --- |
|  |  | **AA** | ***P*-Interaction** | **EPA** | ***P*-Interaction** | **DHA** | ***P*-Interaction** |
| Fish (per 50 g) |  |  |  |  |  |  |  |
| Men | β_2_ (SE) | −0.047 (0.122) | 0.920 | 0.115 (0.026)** | 0.262 | 0.535 (0.102)** | 0.567 |
| Women | β_2_ (SE) | −0.049 (0.160) |  | 0.049 (0.039) |  | 0.422 (0.127)** |  |
| Red meat (per 50 g) |  |  |  |  |  |  |  |
| Men | β_2_ (SE) | 0.575 (0.186)** | 0.482 | −0.050 (0.040) | 0.262 | −0.193 (0.156) | 0.997 |
| Women | β_2_ (SE) | 0.383 (0.286) |  | −0.019 (0.069) |  | −0.378 (0.228) |  |
| Poultry (per 50 g) |  |  |  |  |  |  |  |
| Men | β_2_ (SE) | −0.081 (0.218) | 0.796 | −0.025 (0.047) | 0.002 | 0.122 (0.183) | 0.007 |
| Women | β_2_ (SE) | 0.058 (0.285) |  | 0.194 (0.069) |  | 0.918 (0.227)** |  |
| Soy (per 50 g) |  |  |  |  |  |  |  |
| Men | β_2_ (SE) | −0.056 (0.043) | 0.660 | −0.003 (0.009) | 0.179 | −0.009 (0.036) | 0.749 |
| Women | β_2_ (SE) | −0.027 (0.060) |  | 0.018 (0.015) |  | 0.004 (0.049) |  |
| PUFA intake (en %) |  |  |  |  |  |  |  |
| Men | β_2_ (SE) | −0.035 (0.042) | 0.931 | 0.010 (0.009) | 0.355 | 0.039 (0.036) | 0.414 |
| Women | β_2_ (SE) | −0.027 (0.060) |  | 0.021 (0.015) |  | 0.082 (0.049) |  |
| *n −* 6 PUFA intake (en %) |  |  |  |  |  |  |  |
| Men | β_2_ (SE) | −0.035 (0.045) | 0.948 | 0.011 (0.010) | 0.423 | 0.040 (0.038) | 0.437 |
| Women | β_2_ (SE) | −0.028 (0.063) |  | 0.021 (0.016) |  | 0.084 (0.053) |  |

^1^ β (SE) are regression coefficients (standard errors) from linear regression analysis. Values for the β coefficients represent the percentage change in plasma fatty acid composition for every 50 g per day increment in fish, red meat, poultry and soy consumption or every 1% increment in total energy intake contributed by PUFA. β_2_: model adjusted for age, body mass index, physical activity, total energy intake, alcohol consumption, and other meats or meat alternatives (fish, red meat, poultry, and soy) except in the case for PUFA and *n −* 6 PUFA intake. ^2^ Fatty acid abbreviations: LA (linoleic acid; 18:2 *n −* 6), ALA (alpha-linolenic acid; 18:3 *n −* 3), DGLA (dihomo-gamma-linolenic acid; 20:3 *n −* 6), AA (arachidonic acid; 20:4 *n −* 6), EPA (eicosapentaenoic acid; 20:5 *n −* 3), DHA (docosahexaenoic acid; 22:6 *n −* 3). * *p*-value < 0.05; ** *p*-value < 0.01

**Table S3.** Association between consumption of meat and meat alternatives, polyunsaturated fat (PUFA) and plasma fatty acid composition (weight %) of the Singapore Chinese Health Study participants (*n* = 769) stratified by age (*n* = 367 for age < 60 years, *n* = 402 for age ≥ 60 years) ^1^.

| **Dietary Variables** |  | **Fatty Acid (%)** | | | | | |
| --- | --- | --- | --- | --- | --- | --- | --- |
|  |  | **LA ^2^** | ***P*-Interaction** | **ALA** | ***P*-Interaction** | **DGLA** | ***P*-Interaction** |
| Fish (per 50 g) |  |  |  |  |  |  |  |
| Age < 60 years | β_2_ (SE) | 0.533 (0.381) | 0.496 | 0.022 (0.017) | 0.262 | −0.056 (0.024)* | 0.535 |
| Age ≥ 60 years | β_2_ (SE) | 0.093 (0.349) |  | −0.003 (0.017) |  | −0.030 (0.026) |  |
| Red meat (per 50 g) |  |  |  |  |  |  |  |
| Age < 60 years | β_2_ (SE) | −0.059 (0.612) | 0.939 | −0.061 (0.027)* | 0.972 | 0.021 (0.038) | 0.775 |
| Age ≥ 60 years | β_2_ (SE) | −0.129 (0.569) |  | −0.064 (0.027)* |  | 0.026 (0.043) |  |
| Poultry (per 50 g) |  |  |  |  |  |  |  |
| Age < 60 years | β_2_ (SE) | −0.737 (0.660) | 0.252 | 0.012 (0.029) | 0.478 | 0.006 (0.041) | 0.861 |
| Age ≥ 60 years | β_2_ (SE) | 0.398 (0.637) |  | 0.048 (0.030) |  | 0.009 (0.048) |  |
| Soy (per 50 g) |  |  |  |  |  |  |  |
| Age < 60 years | β_2_ (SE) | −0.068 (0.145) | 0.504 | 0.013 (0.006)* | 0.484 | 0.006 (0.009) | 0.470 |
| Age ≥ 60 years | β_2_ (SE) | 0.098 (0.119) |  | 0.009 (0.006) |  | −0.003 (0.009) |  |
| PUFA intake (en %) |  |  |  |  |  |  |  |
| Age < 60 years | β_2_ (SE) | 0.207 (0.137) | 0.812 | 0.015 (0.006)* | 0.612 | −0.010 (0.009) | 0.804 |
| Age ≥ 60 years | β_2_ (SE) | 0.263 (0.119)* |  | 0.020 (0.006)** |  | −0.006 (0.009) |  |
| n−6 PUFA intake (en %) |  |  |  |  |  |  |  |
| Age < 60 years | β_2_ (SE) | 0.218 (0.146) | 0.791 | 0.016 (0.007)* | 0.616 | −0.010 (0.009) | 0.851 |
| Age ≥ 60 years | β_2_ (SE) | 0.283 (0.127)* |  | 0.021 (0.006)** |  | −0.006 (0.010) |  |

**Table S3.** *Cont.*

| **Dietary Variables** |  | **Fatty Acid (%)** | | | | | |
| --- | --- | --- | --- | --- | --- | --- | --- |
|  |  | **AA** | ***P*−Interaction** | **EPA** | ***P*−Interaction** | **DHA** | ***P*−Interaction** |
| Fish (per 50 g) |  |  |  |  |  |  |  |
| Age < 60 years | β_2_ (SE) | −0.090 (0.139) | 0.887 | 0.077 (0.026)** | 0.871 | 0.454 (0.105)** | 0.837 |
| Age ≥ 60 years | β_2_ (SE) | 0.002 (0.134) |  | 0.091 (0.035)** |  | 0.480 (0.117)** |  |
| Red meat (per 50 g) |  |  |  |  |  |  |  |
| Age < 60 years | β_2_ (SE) | 0.544 (0.223)* | 0.880 | −0.059 (0.041) | 0.221 | −0.259 (0.169) | 0.506 |
| Age ≥ 60 years | β_2_ (SE) | 0.431 (0.219)* |  | −0.013 (0.057) |  | −0.228 (0.191) |  |
| Poultry (per 50 g) |  |  |  |  |  |  |  |
| Age < 60 years | β_2_ (SE) | −0.016 (0.240) | 0.669 | 0.042 (0.044) | 0.269 | 0.213 (0.182) | 0.086 |
| Age ≥ 60 years | β_2_ (SE) | 0.072 (0.245) |  | 0.085 (0.064) |  | 0.676 (0.214)** |  |
| Soy (per 50 g) |  |  |  |  |  |  |  |
| Age < 60 years | β_2_ (SE) | −0.143 (0.053)** | 0.021 | 0.002 (0.010) | 0.924 | −0.010 (0.040) | 0.991 |
| Age ≥ 60 years | β_2_ (SE) | 0.030 (0.046) |  | 0.005 (0.012) |  | 0.003 (0.040) |  |
| PUFA intake (en %) |  |  |  |  |  |  |  |
| Age < 60 years | β_2_ (SE) | −0.078 (0.051) | 0.348 | 0.017 (0.009) | 0.449 | 0.069 (0.039) | 0.446 |
| Age ≥ 60 years | β_2_ (SE) | 0.010 (0.046) |  | 0.010 (0.012) |  | 0.040 (0.041) |  |
| *n −* 6 PUFA intake (en %) |  |  |  |  |  |  |  |
| Age < 60 years | β_2_ (SE) | −0.080 (0.054) | 0.362 | 0.020 (0.010)* | 0.417 | 0.073 (0.041) | 0.434 |
| Age ≥ 60 years | β_2_ (SE) | 0.012 (0.049) |  | 0.010 (0.013) |  | 0.040 (0.044) |  |

^1^ β (SE) are regression coefficients (standard errors) from linear regression analysis.Values for the β coefficients represent the percentage change in plasma fatty acid composition for every 50 g per day increment in fish, red meat, poultry and soy consumption or every 1% increment in total energy intake contributed by PUFA. β_2_: model adjusted for sex, body mass index, physical activity, total energy intake, alcohol consumption, and other meats or meat alternatives (fish, red meat, poultry, and soy) except in the case for PUFA and *n −* 6 PUFA intake. ^2^ Fatty acid abbreviations: LA (linoleic acid; 18:2 *n −* 6), ALA (alpha-linolenic acid; 18:3 *n −* 3), DGLA (dihomo-gamma-linolenic acid; 20:3 *n −* 6), AA (arachidonic acid; 20:4 *n −* 6), EPA (eicosapentaenoic acid; 20:5 *n −* 3), DHA (docosahexaenoic acid; 22:6 *n −* 3). * *p*-value < 0.05; ** *p*-value < 0.01.

**Table S4.** Association between consumption of meat and meat alternatives, polyunsaturated fat (PUFA) and plasma fatty acid composition (weight %) of the Singapore Chinese Health Study participants (*n* = 769) stratified by overweight status (*n* = 394 for BMI < 23 kg/m^2^, *n* = 375 for BMI ≥ 23 kg/m^2^) ^1^.

| **Dietary Variables** |  | **Fatty Acid (%)** | | | | | |
| --- | --- | --- | --- | --- | --- | --- | --- |
|  |  | **LA ^2^** | ***P*-Interaction** | **ALA** | ***P*-Interaction** | **DGLA** | ***P*-Interaction** |
| Fish (per 50 g) |  |  |  |  |  |  |  |
| BMI < 23 kg/m^2^ | β_2_ (SE) | 0.221 (0.371) | 0.757 | −0.006 (0.018) | 0.245 | −0.025 (0.024) | 0.337 |
| BMI ≥ 23 kg/m^2^ | β_2_ (SE) | 0.412 (0.351) |  | 0.025 (0.016) |  | −0.059 (0.026)* |  |
| Red meat (per 50 g) |  |  |  |  |  |  |  |
| BMI < 23 kg/m^2^ | β_2_ (SE) | −0.404 (0.586) | 0.721 | −0.047 (0.028) | 0.915 | 0.009 (0.039) | 0.725 |
| BMI ≥ 23 kg/m^2^ | β_2_ (SE) | 0.308 (0.590) |  | −0.080 (0.026)** |  | 0.035 (0.044) |  |
| Poultry (per 50 g) |  |  |  |  |  |  |  |
| BMI < 23 kg/m^2^ | β_2_ (SE) | 0.458 (0.624) | 0.134 | 0.011 (0.030) | 0.200 | 0.008 (0.041) | 0.873 |
| BMI ≥ 23 kg/m^2^ | β_2_ (SE) | −0.980 (0.683) |  | 0.055 (0.030) |  | −0.008 (0.050) |  |
| Soy (per 50 g) |  |  |  |  |  |  |  |
| BMI < 23 kg/m^2^ | β_2_ (SE) | −0.052 (0.124) | 0.717 | 0.014 (0.006)* | 0.592 | 0.008 (0.008) | 0.163 |
| BMI ≥ 23 kg/m^2^ | β_2_ (SE) | 0.117 (0.137) |  | 0.006 (0.006) |  | −0.008 (0.010) |  |
| PUFA intake (en %) |  |  |  |  |  |  |  |
| BMI < 23 kg/m^2^ | β_2_ (SE) | 0.292 (0.127)* | 0.233 | 0.024 (0.006)** | 0.183 | −0.010 (0.008) | 0.706 |
| BMI ≥ 23 kg/m^2^ | β_2_ (SE) | 0.156 (0.127) |  | 0.011 (0.006)* |  | −0.005 (0.009) |  |
| *n −* 6 PUFA intake (en %) |  |  |  |  |  |  |  |
| BMI < 23 kg/m^2^ | β_2_ (SE) | 0.299 (0.136)* | 0.283 | 0.026 (0.006)** | 0.154 | −0.011 (0.009) | 0.606 |
| BMI ≥ 23 kg/m^2^ | β_2_ (SE) | 0.174 (0.134) |  | 0.012 (0.006)* |  | −0.004 (0.010) |  |

**Table S4.** *Cont.*

| **Dietary Variables** |  | **Fatty Acid (%)** | | | | | |
| --- | --- | --- | --- | --- | --- | --- | --- |
|  |  | **AA** | ***P*−Interaction** | **EPA** | ***P*−Interaction** | **DHA** | ***P*−Interaction** |
| Fish (per 50 g) |  |  |  |  |  |  |  |
| BMI < 23 kg/m^2^ | β_2_ (SE) | 0.115 (0.143) | 0.101 | 0.113 (0.032)** | 0.575 | 0.453 (0.106)** | 0.472 |
| BMI ≥ 23 kg/m^2^ | β_2_ (SE) | −0.200 (0.130) |  | 0.068 (0.030)* |  | 0.510 (0.117)** |  |
| Red meat (per 50 g) |  |  |  |  |  |  |  |
| BMI < 23 kg/m^2^ | β_2_ (SE) | 0.416 (0.225) | 0.921 | −0.039 (0.051) | 0.801 | −0.256 (0.167) | 0.912 |
| BMI ≥ 23 kg/m^2^ | β_2_ (SE) | 0.541 (0.219)* |  | −0.038 (0.050) |  | −0.236 (0.196) |  |
| Poultry (per 50 g) |  |  |  |  |  |  |  |
| BMI < 23 kg/m^2^ | β_2_ (SE) | 0.051 (0.240) | 0.421 | −0.019 (0.054) | 0.023 | 0.309 (0.178) | 0.324 |
| BMI ≥ 23 kg/m^2^ | β_2_ (SE) | −0.160 (0.254) |  | 0.174 (0.057)* |  | 0.640 (0.227)** |  |
| Soy (per 50 g) |  |  |  |  |  |  |  |
| BMI < 23 kg/m^2^ | β_2_ (SE) | −0.051 (0.048) | 0.892 | −0.008 (0.011) | 0.040 | −0.052 (0.035) | 0.015 |
| BMI ≥ 23 kg/m^2^ | β_2_ (SE) | −0.041 (0.051) |  | 0.023 (0.012)* |  | 0.068 (0.137) |  |
| PUFA intake (en %) |  |  |  |  |  |  |  |
| BMI < 23 kg/m^2^ | β_2_ (SE) | −0.064 (0.049) | 0.501 | 0.005 (0.011) | 0.250 | 0.005 (0.037) | 0.073 |
| BMI ≥ 23 kg/m^2^ | β_2_ (SE) | −0.007 (0.048) |  | 0.021 (0.011) |  | 0.102 (0.044)* |  |
| *n −* 6 PUFA intake (en %) |  |  |  |  |  |  |  |
| BMI < 23 kg/m^2^ | β_2_ (SE) | −0.072 (0.053) | 0.417 | 0.005 (0.012) | 0.260 | 0.004 (0.040) | 0.080 |
| BMI ≥ 23 kg/m^2^ | β_2_ (SE) | −0.001 (0.050) |  | 0.022 (0.012) |  | 0.105 (0.046)* |  |

^1^ β (SE) are regression coefficients (standard errors) from linear regression analysis. Values for the β coefficients represent the percentage change in plasma fatty acid composition for every 50 g per day increment in fish, red meat, poultry and soy consumption or every 1% increment in total energy intake contributed by PUFA. β_2_: model adjusted for sex, age, physical activity, total energy intake, alcohol consumption, and other meats or meat alternatives (fish, red meat, poultry, and soy) except in the case for PUFA and *n −* 6 PUFA intake. ^2^ Fatty acid abbreviations: LA (linoleic acid; 18:2 *n −* 6), ALA (alpha-linolenic acid; 18:3 *n −* 3), DGLA (dihomo-gamma-linolenic acid; 20:3 *n −* 6), AA (arachidonic acid; 20:4 *n −* 6), EPA (eicosapentaenoic acid; 20:5 *n −* 3), DHA (docosahexaenoic acid; 22:6 *n −* 3). * *p*-value < 0.05; ** *p*-value < 0.01
